# A global reference database of crowdsourced cropland data collected using the Geo-Wiki platform

**DOI:** 10.1038/sdata.2017.136

**Published:** 2017-09-26

**Authors:** Juan Carlos Laso Bayas, Myroslava Lesiv, François Waldner, Anne Schucknecht, Martina Duerauer, Linda See, Steffen Fritz, Dilek Fraisl, Inian Moorthy, Ian McCallum, Christoph Perger, Olha Danylo, Pierre Defourny, Javier Gallego, Sven Gilliams, Ibrar ul Hassan Akhtar, Swarup Jyoti Baishya, Mrinal Baruah, Khangsembou Bungnamei, Alfredo Campos, Trishna Changkakati, Anna Cipriani, Krishna Das, Keemee Das, Inamani Das, Kyle Frankel Davis, Purabi Hazarika, Brian Alan Johnson, Ziga Malek, Monia Elisa Molinari, Kripal Panging, Chandra Kant Pawe, Ana Pérez-Hoyos, Parag Kumar Sahariah, Dhrubajyoti Sahariah, Anup Saikia, Meghna Saikia, Peter Schlesinger, Elena Seidacaru, Kuleswar Singha, John W Wilson

**Affiliations:** 1 International Institute for Applied Systems Analysis (IIASA), Laxenburg, Austria; 2 Université catholique de Louvain (UCL)—Earth and Life Institute, Louvain-la-Neuve, Belgium; 3 European Commission—Joint Research Centre (JRC), Ispra, Italy; 4 Karlsruhe Institute of Technology (KIT), Department of Atmospheric Environmental Research, Garmisch-Partenkirchen 82467, Germany; 5 Vlaamse Instelling voor Technologisch Onderzoek (VITO), Mol, Belgium; 6 COMSATS Institute of Information Technology, Islamabad, Pakistan; 7 Pakistan Space and Upper Atmosphere Research Commission (SUPARCO), Islamabad, Pakistan; 8 Gauhati University, Guwahati, India; 9 Taguay, Córdoba, Argentina; 10 Instituto de Clima y Agua, Instituto Nacional de Tecnología Agropecuaria (INTA), Buenos Aires, Argentina; 11 Dipartimento di Scienze Chimiche e Geologiche, University of Modena and Reggio Emilia, Modena, Italy; 12 Lamont-Doherty Earth Observatory of Columbia University, Palisades, New York, USA; 13 The Earth Institute, Columbia University, New York, USA; 14 The Nature Conservancy, New York, USA; 15 Institute for Global Environmental Strategies, Kamiyamaguchi, Hayama, Japan; 16 Vrije Universiteit, Amsterdam, Netherlands; 17 Politecnico di Milano, Milano, Italy; 18 B.Borooah College, Guwahati, 781007 India; 19 Don Bosco College of Engineering and Technology, Guwahati, India; 20 The Tropical Agriculture Research and Higher Education Center (CATIE), Turrialba, Costa Rica; 21 University of Idaho, Moscow, USA; 22 TomTom, Amsterdam, Netherlands; 23 Department of Zoology and Entomology, University of Pretoria, Pretoria, South Africa

**Keywords:** Environmental chemistry, Agriculture, Sustainability, Geography

## Abstract

A global reference data set on cropland was collected through a crowdsourcing campaign using the Geo-Wiki crowdsourcing tool. The campaign lasted three weeks, with over 80 participants from around the world reviewing almost 36,000 sample units, focussing on cropland identification. For quality assessment purposes, two additional data sets are provided. The first is a control set of 1,793 sample locations validated by students trained in satellite image interpretation. This data set was used to assess the quality of the crowd as the campaign progressed. The second data set contains 60 expert validations for additional evaluation of the quality of the contributions. All data sets are split into two parts: the first part shows all areas classified as cropland and the second part shows cropland average per location and user. After further processing, the data presented here might be suitable to validate and compare medium and high resolution cropland maps generated using remote sensing. These could also be used to train classification algorithms for developing new maps of land cover and cropland extent.

## Background & Summary

The spatial extent of cropland has been mapped from remote sensing via many different initiatives as part of global land cover mapping activities, e.g., GLC-2000^1^, MODIS land cover^2^, GlobeLand30^3^ and the Climate Change Initiative (CCI) of the European Space Agency^4^. Despite the availability of these and many other products, large spatial disagreement on the location and distribution of cropland still exists^5–7^.

As such, quality-assured reference data are needed to undertake robust quantitative assessments and detailed comparisons of global products regarding their representation of cropland extent. Reference data sets can be collected *in-situ*, e.g., the Land Use Cover Area frame Sample (LUCAS) across EU member states^8^, but due to the high costs involved in field surveys, they are more often gathered through interpretation of high or very high resolution satellite imagery. Some of the reference data used to validate different global land cover products are now being made openly available, e.g., through the GOFC-GOLD validation portal^9^. Because these data sets are not specifically tailored to cropland validation, sample sizes are insufficient, making their efficacy in quality assessments questionable, especially given the lack of sensitivity of accuracy indices^7^.

**Figure 1:**
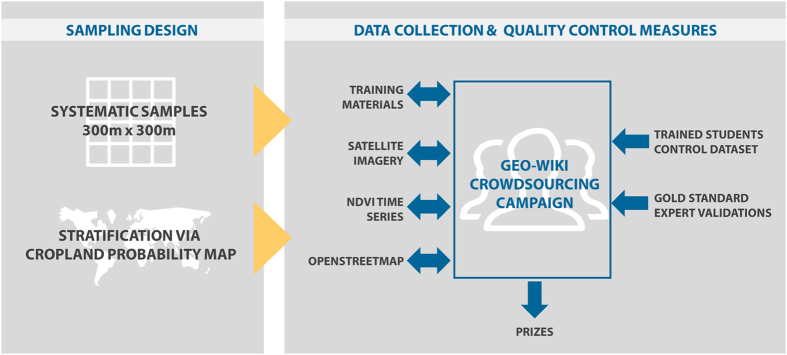
Schematic representation of the design and implementation of the crowdsourcing campaign to collect reference samples designed for cropland map validation, implemented using the Geo-Wiki (http://www.geo-wiki.org/) crowdsourcing tool.

To collect reference samples specifically designed for cropland map validation, we conducted a three-week cropland identification campaign during September, 2016. The campaign was implemented using the Geo-Wiki (http://www.geo-wiki.org/) crowdsourcing tool. A schematic showing the design and implementation of the campaign is illustrated in Fig. 1. A secondary motivation of the campaign was to gain a better understanding of crowdsourced data quality as well as the reasons why volunteers participate in crowdsourcing campaigns.

This campaign builds on previous crowdsourcing campaigns using Geo-Wiki^10^, e.g., to validate a map of land availability for biofuels^11^ and to map wilderness globally^12^, while the results from several campaigns were used to produce a global hybrid cropland map^13^, among others. The scope of the early campaigns was generally directed towards improving global land cover and land use reference data^10^, whereas the campaign described here focuses specifically on cropland data. In addition to validation, the data presented here also represent a valuable training tool that can be used to develop new land cover or cropland extent maps as well as to train algorithms to produce remote sensing-based products^14,15^.

## Methods

To develop the cropland validation campaign, cropland *per se* had to be defined, and a sample of systematically selected areas was generated. At the same time, the Geo-Wiki platform was modified to implement the campaign, the incentive scheme was developed, and the control data for quality assurance were collected. This section describes the main components of the campaign as outlined in Fig. 1.

### Cropland definition

In order to distinguish cropland from other classes, the definition used for the campaign follows that of GEOGLAM/JECAM^16,17^ in which ‘The annual cropland from a remote sensing perspective is a piece of land of a minimum of 0.25 ha (minimum width of 30 m) that is sowed/planted and harvestable at least once within the 12 months after the sowing/planting date. The annual cropland produces an herbaceous cover and is sometimes combined with some tree or woody vegetation’. According to this GEOGLAM/JECAM definition, perennial crops, agroforestry plantations, palm oil, coffee, tree crops and fallows are not included in the cropland class. The following exceptions to this definition were made:

- Sugarcane plantations and cassava crops are included in the cropland class, although they have a longer vegetation cycle and are not planted yearly.
- Taken individually, small plots, such as legumes, do not meet the minimum size criterion of the cropland definition. However, when considered as a continuous heterogeneous field, they are included in cropland.

Moreover, greenhouse crops cannot be monitored by remote sensing and are thus excluded from the definition. Note that the use of this definition may lead to underestimation of cropland in the situation where legumes or other crops are planted among tree crops such as fruit and nut trees or where fields were fallow for 1 or more years but still cultivated. This would not be picked up in the visual interpretation of the imagery using Geo-Wiki although the use of Google Earth historical imagery and the Normalized Difference Vegetation Index (NDVI) profiling tool may have helped to identify cropland in the latter situation.

### Sampling design

A stratified systematic sampling procedure was applied to generate the sample locations where the validation would take place in frames/cells of 1°×1° (geographic coordinate system with latitude and longitude) across the globe. A given replicate corresponds to a relative location in each frame. The scheme was designed to correct the distortion of the non-equal area projection. These cells serve as an instrument for defining a first-phase sample.

The strata used were derived from the IIASA cropland probability map^13^ with the aim of sampling areas of lower or higher probability of misclassification with different rates. Areas with a cropland probability between 25 and 75% were assumed to be more difficult to classify and were therefore sampled with a higher rate, while areas with very low or very high probability of cropland were sampled at a lower rate as they are easier to classify. Table 1 summarizes the strata and distribution of samples in each stratum. The size for each stratum as well as the calculated weights that should be used for accuracy metrics are also shown.

The sampling unit was a frame/pixel of 300 m×300 m corresponding to the grid of PROBA-V images and the final number of sampling units was 35,866.

### Data collection using Geo-Wiki

**Figure 2:**
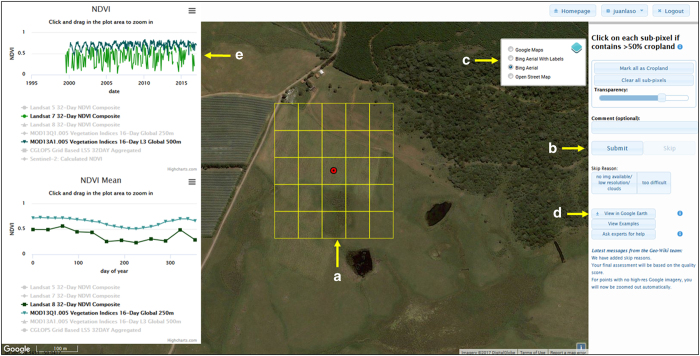
The Geo-Wiki interface (http://www.geo-wiki.org) for collecting cropland information based on image interpretation. (**a**) is the sub-grid of pixels that users must classify; (**b**) is the Submit button that users must press once they have completed their interpretation; (**c**) allows the user to change the background imagery; (**d**) shows the ‘View in Google Earth’ button, which users can press to be shown the location in Google Earth so that that they can view historical imagery; and (**e**) shows the NDVI profiles that can be viewed when the user clicks on a location.

**Figure 3:**
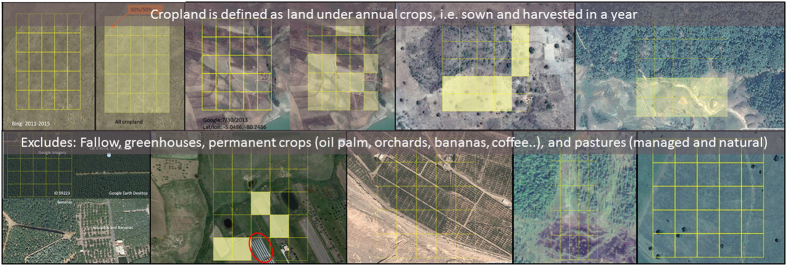
Definition and examples of cropland (yellow shading) and areas of non-cropland as shown in a gallery of examples on Geo-Wiki (http://www.geo-wiki.org/Application/modules/sigma_validation/sigma_gallery.html).

The reference data were acquired through a dedicated Geo-Wiki interface (Fig. 2). Once a participant was registered and logged on, he/she could see a sample location where a semi-transparent 300×300 m frame subdivided in 25 grid cells is superimposed on Google Maps imagery (indicated by A in Fig. 2). Users were then asked to click (i.e., shade in yellow) all grid cells covered by more than 50% cropland. Thus, the final values for sampling units (i.e., a 300×300 m frame=one location) were cropland proportions ranging from 0 (absence of cropland) to 100%. When all sub-cells were examined, the user could either click the submit button or the skip button (indicated by B in Fig. 2) and was then shown the next randomly selected sample location. The user could also add comments regarding the observed location and then submit the validation. The cropland definitions were provided to the participants in an introductory video and through an info button in the Geo-Wiki interface. Additional tools and learning materials were provided to the participants to aid their interpretations. For example, in Geo-Wiki it is possible to switch between imagery from Google Maps and Microsoft Bing as well as viewing the location on OpenStreetMap (indicated by C in Fig. 2), which can provide additional useful information. The system registers whether a participant used imagery from Google Maps or not, which is included as a variable in the data set. Any location could also be saved as a keyhole markup language (kml) file for visualization using the desktop version of Google Earth (indicated by D in Fig. 2), which provides historical imagery, 3D viewing capabilities, geotagged photographs from Panoramio, etc. The usage of this feature was also registered in the data set. Participants were asked to use imagery from the latest date possible between Google Maps and Bing. Learning materials were compiled into an online gallery (Fig. 3), which provided the participants with different examples of cropland and non-cropland surfaces (http://www.geo-wiki.org/Application/modules/sigma_validation/sigma_gallery.html). Finally, it is possible to view different time series of vegetation indices, e.g., the NDVI (indicated by E in Fig. 2), obtained from different satellite sensors, i.e., Landsat 7, 8, MODIS and PROBA-V. These indices allowed participants to view the profiles of vegetation change over time at a particular location, which could help with satellite image interpretation, e.g., cropland is often characterized by a rapid increase in NDVI at growing stage after planting and a rapid decline near maturity stage or after harvesting.

Feedback was provided to participants as the campaign progressed using the Geo-Wiki Facebook page https://www.facebook.com/GeoWiki, which contained additional examples and a link to the YouTube explanatory video https://youtu.be/PR3xMPPyp-I showing how to use the interface. Participants could request help from experts for images that were difficult to classify and the answers were then posted to Facebook for all to view.

### Quality control measures

Out of the total sample locations, 2,000 were randomly selected and validated by a group of three students trained in satellite imagery interpretation. The methodology for validation of control points was the same as for normal locations. These sample locations were compared for consistency, resulting in the removal of 207 sample units where there was disagreement in 3 or more grid cells/sub-pixels between the student validators. Additionally, independent verification was undertaken by experts at the International Institute for Applied Systems Analysis (IIASA) to ensure the quality of the control data set. Experts are members of IIASA staff with a background in remote sensing or geospatial sciences and considerable experience in image classification. This control data set was then used during the campaign, where participants received one control location for every 20 sample locations although this control location could appear at any point during the sequence of 20 samples. Each time a control location was viewed, the submission sent by the participant was compared with the control validation and a quality score was calculated for each participant as shown in Table 2. This, in combination with the amount of validations undertaken, was used to determine the participant’s ranking on the campaign leader board.

The campaign aimed to validate all sample locations at least 3 times by different participants. The final result achieved was that the majority of locations (32,287) were validated 4 to 7 times. Control points were validated more often, sometimes more than once by the same person to check for consistency. Despite a technical problem in the middle of the campaign, where some validations done in the middle of the campaign were not recorded, the full sample of validations was obtained.

### Incentives and motivations

The top 30 participants (ranked by quality score) had the option to choose between becoming a co-author on a scientific paper or receiving an Amazon gift voucher ranging in value from 50 to 750 EUR (Table 3) depending upon the final position on the campaign leader board. A total of 26 participants chose to be co-author. They were also asked to fill out a survey providing some basic information about themselves and details regarding their motivation in participating in the campaign.

The same survey as that sent to the top 30 was also sent to the other participants where they were offered the following incentive: they were entered into a draw in which they could win one of two Amazon vouchers of €50 euros. A total of 20 additional answers were received.

From the 1,793 control locations, a further sub-sample of 60 locations was selected and then evaluated independently by three land cover experts at IIASA following the same methodology as a normal participant. These locations were then reviewed for consensus between the experts, creating a gold standard data set. Although the gold standard was not used to calculate the quality score, it is provided here as an additional data set for independent quality and reliability assessment. These 60 locations were evaluated by all participants sequentially in the middle of the campaign, although no notice was given to the participants and no changes to the Geo-Wiki interface were made.

## Data Records

The data are presented in six different data records. The first three data records contain all of the grid cells marked as cropland by either the campaign participants (Data record 1, *n*=1,086,485), the controls from the trained students (Data Record 2, *n*=8,918) or the gold standard (Data record 3, *n*=582) and can be found in crop_all.txt, crop_con.txt and crop_exp.txt (Data Citation 1), respectively. The format and information contained in these first three data records is shown in Table 4. Note that when these data correspond to the control data or to data from the experts, the following fields are not present: comment, timestamp, used_gmaps, viewed_ge, and skip_reason. The userid field in Data Record 2 is the number 111,111 and Data Record 3 is the number 222,222.

Additionally, data records 4 to 6 show the information compiled per 300 m×300 m frame and per user, i.e., one record shows the average (mean) cropland from the 25 grid cells from a given user at a given location. Data Record 4 (*n*=203,515) contains data from all participants, data record 5 (*n*=1,793) contains the control data from the trained students while Data Record 6 contains the expert data (*n*=60). These data sets can be found in loc_all.txt, loc_con.txt and loc_exp.txt (Data Citation 1), respectively, while the format and field descriptions are provided in Table 5. As in data sets 2 and 3, the userid field in data record 5 is the number 111,111 and in data record 6 it is the number 222,222.

## Technical Validation

**Figure 4:**
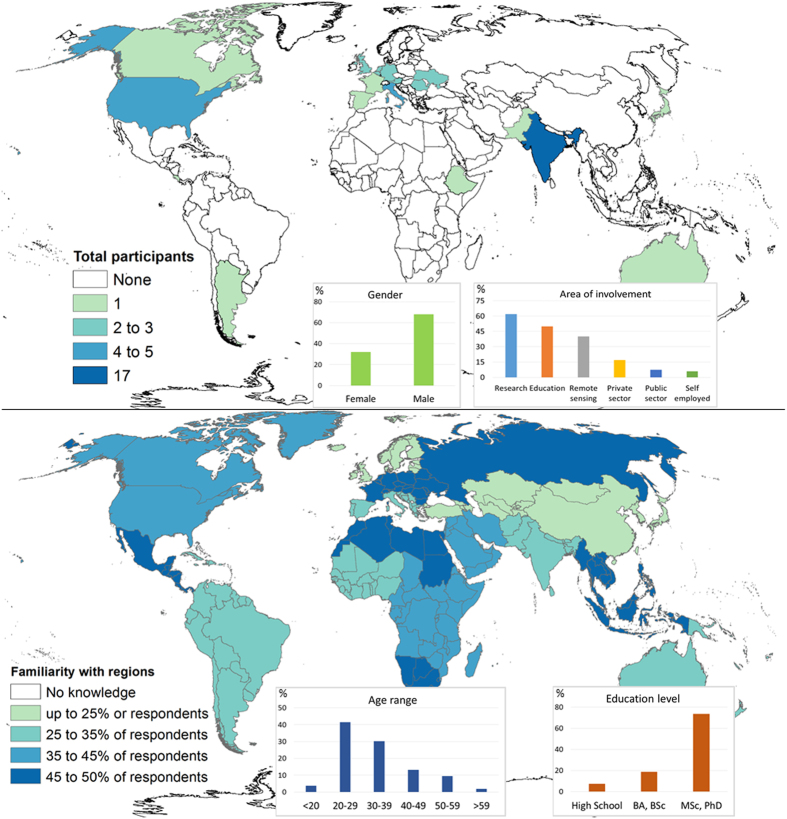
Geographical location, previous knowledge and general information from the participants who filled in the survey at the end of the cropland validation campaign (*n*=50).

Figure 4 illustrates the origin of the 50 participants who provided information on the post-campaign survey and their familiarity with the regions validated as well as general information. It is clear that the majority of participants were male (68%) with a background in research (62%), highly educated (92%), and between 20 and 39 years of age (72%). The largest number of participants were from India (17) although more than 20 countries were represented. Participants had varying knowledge of different parts of the world although there was no area where participants had zero familiarity. This may reflect the geographical spread of the participants and their backgrounds.

**Figure 5:**
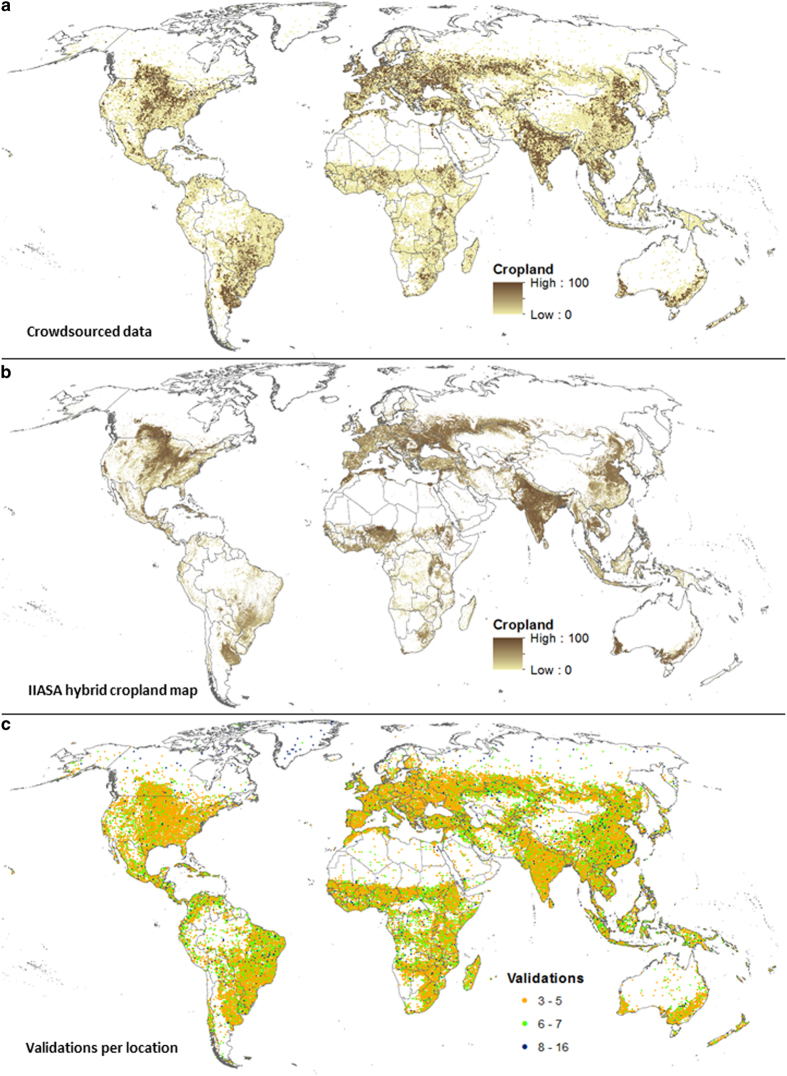
Cropland validation campaign and worldwide spatial distribution of cropland. The (**a**) presents cropland data collected during the cropland validation campaign, showing the mean cropland percentage per location and on the (**b**) the IIASA-IFPRI hybrid cropland map is shown for comparison. The third (**c**) shows the number of validations at each location during the campaign.

Figure 5a shows data collected during the campaign, expressed as the average (mean) cropland percentage per location and its global distribution. Figure 5b contains the IIASA-IFPRI hybrid cropland percentage map^13^, and it is provided as a reference; in general, the patterns of cropland between the two maps are similar. Figure 5c shows the number of times a location was validated, where the majority of locations were classified at least 3 to 5 times.

## Usage Notes

The primary use of this reference data set is to validate global cropland maps generated using remote sensing that range from 60 to 300 m in resolution. More specifically, the data allows for an extensive spatially explicit validation of the cropland layer due to the rich amount of reference data. A validation exercise is planned for a 300 m cropland map that has been created for agricultural monitoring purposes as part of the FP7-funded SIGMA project (http://www.geoglam-sigma.info/). The data can also be used to train classification algorithms in developing new cropland maps based on remote sensing or to create hybrid cropland maps by fusing together existing cropland products^13^. Finally, it would be possible to use the data for studies about the quality of crowdsourced data.

## Additional Information

**How to cite this article:** Laso Bayas, J.C. *et al.* A global reference database of crowdsourced cropland data collected using the Geo-Wiki platform. *Sci. Data* 4:170136 doi: 10.1038/sdata.2017.136 (2017).

**Publisher’s note:** Springer Nature remains neutral with regard to jurisdictional claims in published maps and institutional affiliations.

## Supplementary Material



## Figures and Tables

**Table 1:** Strata, sample distribution and strata sizes in the cropland validation campaign.

**Stratum (% cropland probability)**	**Number of samples**	**Percent share**	**Stratum size (Million km**^**2**^)	**Weights (size of one sample, km**^**2**^)
1 (0%)	500	1.39	84.01	168026
2 (0–25%)	10960	30.56	18.76	1712
3 (25–75%)	15984	44.57	14.66	917
4 (>75%)	8422	23.48	16.47	1955
Calculated weights needed for computing accuracy indexes are also shown.				

**Table 2:** Quality score calculation per location. Units for agreement are in number of grid cells/sub-pixels per 300 m×300 m location.

**Agreement with control**	**Points to the participant**	**Agreement with control**	**Points to the participant**
25	25	12	−1
24	23	11	−3
23	21	10	−5
22	19	9	−7
21	17	8	−9
20	15	7	−11
19	13	6	−13
18	11	5	−15
17	9	4	−17
16	7	3	−19
15	5	2	−21
14	3	1	−23
13	1	0	−25

**Table 3:** Financial rewards offered according to the final ranking of the participants.

**Rank**	**Proposed financial prize**
1	€ 750
2	€ 500
3	€ 300
4	€ 100
5	€ 85
6	€ 65
7–9	€ 50
10–30	€ 25

**Table 4:** The format and field descriptions of data records containing all grid cells marked as cropland.

**Variable**	**Type**	**Description**	**Example**
location_id	Numeric, continuous	Unique number identifying each location in the campaign.	47286
userid	Numeric, continuous	Numeric field used to uniquely identify participants/users	11182
sub_id	Numeric, continuous	Sequentially assigned number identifying every submission done in the system	383725
comment	Text	Comments entered by the participant	Apparent pastures
timestamp	Date and time	Exact time and date when the submission was entered into the system	2016-09-16 13:20:19
used_gmaps	Yes=‘t’No=‘f’	Registers whether the participant was viewing the Google background imagery when the submission was done	t
viewed_ge	Yes=‘t’No=‘f’	Registers whether the participant pressed the button labelled View in Google Earth	f
skip_reason	Numeric, categorical	Registers whether the participant did not skip the point (**Skip=0**), skipped the point and used the reason ‘no img. available/ low resolution/ clouds’ (**Skip=1**), or skipped the point and used the reason ‘too difficult’ (**Skip=2**)	0
sub_item_id	Numeric, continuous	Unique identifier of each grid cell classified as cropland at a given location by a given user	10579829
sub_item_x	Numeric, continuous	Longitude of each grid cell centroid inside a frame/location (decimal degrees)	30.95357144
sub_item_y	Numeric, continuous	Latitude of each grid cell centroid inside a frame/location (decimal degrees)	−20.75119048

**Table 5:** Format and field descriptions of data records containing average (mean) cropland per frame/location and user.

**Variable**	**Type**	**Description**	**Example**
location_id	Numeric, continuous	Unique number identifying each location in the campaign.	47286
userid	Numeric, continuous	Numeric field used to uniquely identify the participants/users.	5
sumcrop	Numeric, continuous	Average (mean) cropland at a given location in percentage	80
loc_cent_X	Numeric, continuous	Longitude of a frame/location centroid (decimal degrees)	−39.75
loc_cent_Y	Numeric, continuous	Latitude of a frame/location centroid (decimal degrees)	−8.047619048
